# The efficacy and safety of temperature controlled dual-mode radiofrequency in women with vaginal laxity

**DOI:** 10.1186/s12905-023-02261-y

**Published:** 2023-03-23

**Authors:** Lixia FU, Senyang Long, Qin LI, Hainan XU, Ling Guo, Huarong Wang, Zhongyan Zheng, Jing Zhang

**Affiliations:** 1 Children’s Central Hospital, School of Medicine, University of Electronic Science and Technology of China, No. 1617, Riyue Avenue, Qingyang District, 611731 Chengdu, Sichuan Province China; 2 Department of Obstetrics and Gynecology, Pelvic Floor Disease Diagnosis and Treatment Center, Shengjing Hospital of China Medical University, Shenyang City, Liaoning Province China

**Keywords:** Vaginal laxity, Temperature controlled RF, Sexual function

## Abstract

**Objective:**

Vaginal laxity could negatively influence women’s sexual function. This study aimed to explore the efficacy and safety of temperature controlled dual-mode (monopolar and bipolar) radiofrequency (RF) in women with vaginal laxity.

**Methods:**

A total of 102 patients with vaginal laxity were treated with temperature-controlled RF. The present study implemented Vaginal Laxity Questionnaire (VLQ), Female Sexual Function Index (FSFI) questionnaire and Sexual Satisfaction Questionnaire (SSQ) on all patients at baseline and after treatment. Pelvic Organ Prolapse Quantification System (POP-Q) system was applied to physical examination, and vaginal manometer to examine the strength of voluntary contractions of the pelvic floor muscles.

**Results:**

The VLQ score was gradually increased after RF treatment at 1, 3, 6 and 12 months, accompanying by the significant improvement in total FSFI scores and the six domains (sexual desire, sexual arousal, lubrication, orgasm, satisfaction, pain). The increased sexual satisfaction based on the SSQ score was found after temperature-controlled RF. The result of POP-Q stage showed significant difference in women after treatment, with the women having Stage I of 45.10% at baseline, 36.27% at 1 month, 28.43% at 3 months, 19.61% at 6 months and 10.78% at 12 months. The mean pressure and mean duration of pelvic contractions were increased gradually at the 1-, 3-, 6- and 12- month follow-up.

**Conclusion:**

Temperature controlled dual-mode (monopolar and bipolar) radiofrequency may be associated with improvement of vaginal laxity, and contribute to enhancement to female sexual function and pelvic floor muscles.

## Introduction

As we known, potential consequences were associated with vaginal delivery that extended beyond the postpartum period are urinary incontinence (UI), pelvic organ prolapse (POP), chronic pelvic pain (CPP), and vaginal laxity [1, 2]. International Urogynecological Association (IUGA)/International Continence Society (ICS) defined vaginal laxity as complaint of excessive vaginal flaccidity, and married women were at higher risk of vaginal laxity [3]. Vaginal laxity was induced by a variety of factors including pregnancy, vaginal delivery and connective tissue changes caused by aging [4, 5]. Vaginal laxity mainly influenced women’s sexual health [6] and quality of life [7] due to typical symptoms including pelvic organ prolapse, stress incontinence, and overactive bladder syndrome. Little was known concerning the incidence of vaginal laxity but approximately 38% of 2,621 women were reported to suffer from vaginal laxity in an electronic Personal Assessment Questionnaire-Pelvic Floor [8]. The first based drug prevention study by Schiavi MC et al. demonstrated that non-pharmacological oral therapy (a combination of hyaluronic acid, chondroitin sulfate, curcumin, and quercetin) achieved subjective improvements in patient well-being and sexual life in reproductive age [9].

A plenty of therapies were available against vaginal laxity, including surgical and non-surgical procedures. Surgical interventions are represented by minimally invasive surgical approaches such as vaginal tightening surgery (vaginoplasty and perineoplasty) [10], which were most commonly preferred clinically. In addition to achievement of satisfactory results such as the improvement on vaginal introitus tightness, surgical options may also increase pain intensity and prolong postoperative recovery time [11–13], which were associated with increased risks of nerve damage and sensation loss [14, 15]. The strength of perineum muscles can be enhanced by non-surgical procedures such as Kegel exercises and electrical stimulation [16].

As one of the energy devices, non-ablative radiofrequency (RF) is an electromagnetic wave that generated heat when encountering tissue impedance, resulting in restoring connective tissue and tightening tissue [17]. It has been frequently applied to cosmetic dermatology, including facial and body rejuvenation, and has achieved optimal outcomes in smoothing irregular body texture, reducing skin relaxation and shortening recovery time [18, 19]. RF contributed to improvement on tightness of the vaginal canal and sensitivity of vulvovaginal tissues through inducing procollagen proliferation, neoelasticity and neovascularization in submucosa, and it was widely used to treat vulvovaginal atrophy (VVA), genitourinary syndrome of menopause (GSM), orgasmic dysfunction and stress urinary incontinence (SUI) [14]. Compared to laser, RF had an additional advantage in protecting vaginal tissues due to electromagnetic wave heat generated through the tissue impedance in vaginal tissue [20, 21]. RF had some positive effects on stress urinary incontinence [22], vaginal laxity [14], skin rejuvenation [23], headache [24], and cardiac arrhythmias [25]. In this study, a temperature controlled dual-mode (monopolar and bipolar) RF was applied to patients with vaginal laxity, and its efficacy and safety were evaluated to determine the treatment satisfaction.

## Method

### Sample size

The sample size was estimated using difference between two dependent means (matched pairs) in G*Power software (3.1.9.2), with type I error α of 0.05, type II error β of 0.30, and power of 0.9. The total sample size was determined to be 97 participants. One hundred and two subjects were enrolled to cover 5% dropout.

### Study population

A total of 102 women who presented with symptoms of vaginal laxity was enrolled in this study according to the exclusion and inclusion criteria. Inclusion criteria: (1) females aged 25 ~ 48 years old with at least 1 delivery; (2) the score of Vaginal Laxity Questionnaire (VLQ) ≤ 3 [defined as very loose (score = 1), moderately loose (score = 2), slightly loose (score = 3)] [14]; (3) the score of Female Sexual Function Index (FSFI) ≤ 26.55 [26]; (4) Pelvic Organ Prolapse Quantification System (POP-Q) stages 0–1 (suggesting normal pelvic support) [27], and (5) patients had sexual life at least once a month with a regular male partner. Exclusion criteria: patients with congenital reproductive tract abnormalities, previous surgery or intervention for vagina, vaginal bleeding, vaginitis or other infectious disease (e.g. genital herpes), implanted medical devices or copper intrauterine device, genital fistula or thin rectovaginal septum, serious diseases of the genitourinary system (e.g. cervical cancer), other malignancies, serious diseases of other organs (such as heart, brain, kidney) or mental illness; disorders of consciousness and communication, and oral drugs to affect sexual functions. Research involving human participants, human material, or human data, were performed in accordance with the Declaration of Helsinki. Ethics Committee of Chengdu Women’s and Children’s Central Hospital approved the study. This was a retrospective study, so informed consent was waived.

### Treatment procedure with temperature-controlled dual-mode (monopolar and bipolar) RF

Temperature-controlled RF was applied firstly in the monopolar mode with a set temperature of 40 ~ 45 °C at 35 ~ 40 watts. The whole vaginal wall was divided into 3 regions according to the upper vaginal wall at the 12 o’clock position, and 3 regions were slowly rubbed with the vaginal probe for 5 min, respectively. Another RF treatment was performed in the bipolar mode for 10 min (5 min for each region) with temperatures maintained between 40 and 45 °C at 35 ~ 40 W. Within a region, we rotated the vaginal probe in the clockwise direction around the axis of the probe, and a coupling gel was used to maintain adequate contact between the vaginal probe and vaginal mucosa. Each patient underwent five RF sessions, with an interval of 15 days between them. After treatment, the patients were allowed to return to normal activities, they were recommended stopping sexual intercourse 48 h after the RF session in case of intravaginal treatment. The outcomes of all the subjects were observed at baseline, as well as at 1 month, 3 months, 6 months and 12 months.

### Self-assessment of vaginal laxity by VLQ

VLQ, as a 7-point Likert scale, was performed to evaluate self-assessment of vaginal laxity. The scores were positively correlated with tightness of vaginal laxity. Score 1 ~ 7 was defined as very loose, moderately loose, slightly loose, neither loose nor tight, slightly tight, and moderately tight or very tight, respectively. VLQ score greater than 4 was described as “no vaginal laxity”.

### The female sexual function index (FSFI) questionnaire

FSFI questionnaire is important in sexology, gynecology, and venereology [28], which consists of six domains related to sexual activity (sexual desire, sexual arousal, lubrication, orgasm, satisfaction, pain) with the total score from 2.0 to 36.0 points (Table 1), and a score lower than 26.55 was considered to indicate the risk of a sexual dysfunction [29].

**Table 1:** Female Sexual Function Index (FSFI) questionnaire included six domains related to sexual activity

Six domains	Questions number	Scores
Sexual desire	1 ~ 2	1 ~ 5
Sexual arousal	3 ~ 6	0 ~ 5
Lubrication	7 ~ 10	0 ~ 5
Orgasm	11 ~ 13	0 ~ 5
Satisfaction	14 ~ 16	0 ~ 5
Pain	17 ~ 19	0 ~ 5

### Sexual satisfaction questionnaire (SSQ)

The SSQ questionnaire for the level of sexual satisfaction consists of 10 statements [30]. The respondents address these statements, with a 4-point Likert scale from strongly disagree to strongly agree. Higher scores reflected higher satisfaction. The theoretical, distribution of scores is within the 10 ~ 40 range.

### Physical examination was performed using the POP-Q system

The following indexes were recorded using the POP-Q system, including point Aa (midline point of anterior vaginal wall 3 cm and corresponds to the ureterovesical crease), point Ba (the most distal position of the anterior vaginal wall), point C (most distal/dependent edge of cervix or vault), point D (location of posterior fornix), point Ap (point on midline posterior vaginal wall 3 cm proximal to hymenal ring), point Bp (the most distal position of the posterior vaginal wall), perineal body (Pb), genital hiatus (GH), and total vaginal length (TVL).

### Measurement of the strength of voluntary contractions of the pelvic floor muscles

The strength of voluntary contractions of the pelvic floor muscles was examined based on the average pressure (mmHg) by vaginal manometer, as well as total duration (seconds) according to 3 consecutive pelvic floor muscle contractions.

### Statistical analyses

Data were calculated by using SPSS 22.0 using paired Student’ s t test or *χ*^2^ test, adopting a significance level of 5% (*P <* 0.05). Categorical variables were presented as frequency (percentage). After using Kolmogorov-Smirnov test to analyze the normality, the continuous variables with normal distribution were showed as mean ± standard deviation (SD).

## Result

### Patients and baseline characteristics

The baseline characteristics of the included subjects were listed in Table 2. The patients (n = 102) had the age range of 27 ~ 48 (mean: 37.37 ± 6.80 years) with body mass index (BMI) ranged from 18.05 to 23.92 kg/m^2^ (mean: 20.99 ± 1.65 kg/m^2^). A total of 54 women had a mild labor intensity, and 48 cases had a moderate labor intensity. The gravidity and parity of all subjects were 1.65 ± 0.77 and 1.28 ± 0.47, respectively. The average maximum fetal weight was 3.56 ± 0.22 kg (it ranged from 3.21 to 3.99 kg).

**Table 2:** Sociodemographic characteristics of the study subjects

Parameters	Value
Age (years)	37.37 ± 6.80 (27 ~ 48)
Nature of work	
Mental work	50
Physical labor + Mental work	52
Labor intensity	
Mild	54
Moderate	48
Disease duration (years)	3.43 ± 2.24 (0.1 ~ 7.9)
Body mass index (BMI, kg/cm^2^)	20.99 ± 1.65 (18.05 ~ 23.92)
Gravidity	1.65 ± 0.77 (1 ~ 5)
Parity	1.28 ± 0.47 (1 ~ 3)
Maximum fetal weight (kg)	3.56 ± 0.22 (3.21 ~ 3.99)

### The improved vaginal laxity in women after treated with temperature controlled dual-mode RF

**Fig. 1:**
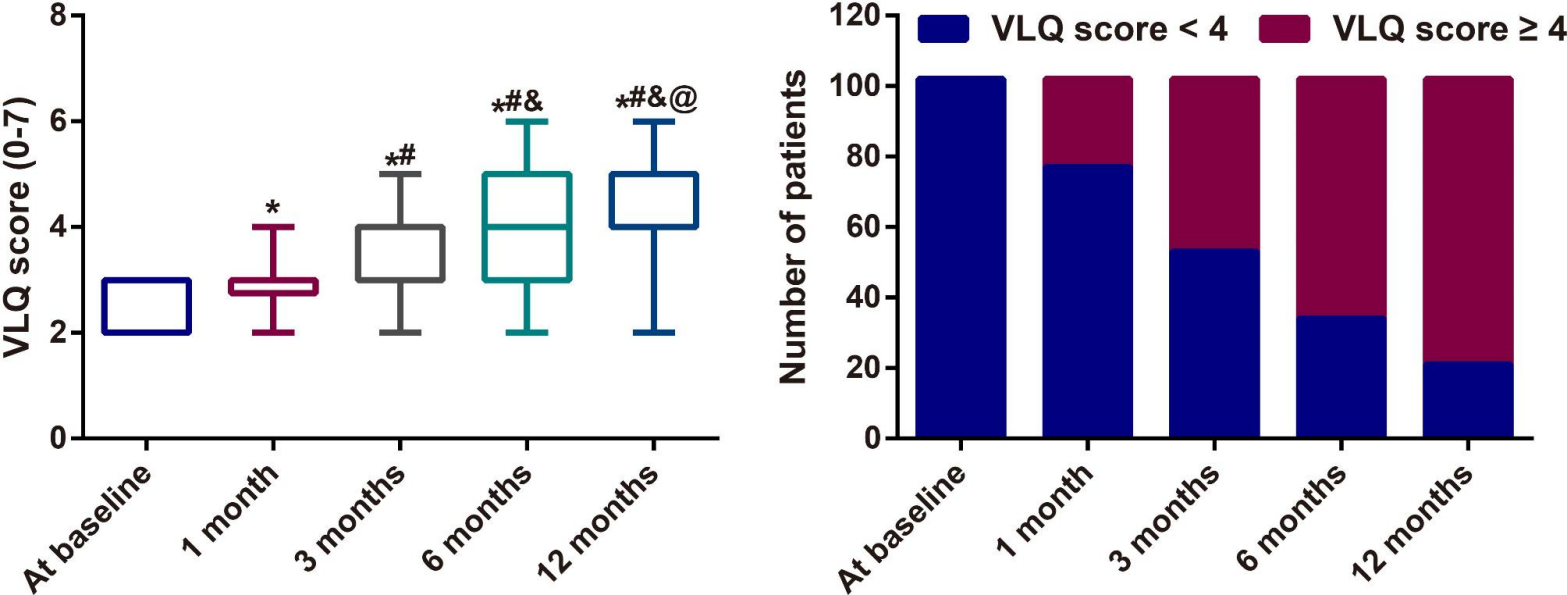
The improved vaginal laxity using Vaginal Laxity Questionnaire (VLQ) scores in women (n = 102) at 1 month, 3 months, 6 months and 12 months after temperature controlled dual-mode radiofrequency (RF) Note: * *P <* 0.05 as compared VLQ score at baseline; # *P <* 0.05 as compared VLQ score at 1 month after treatment; & *P <* 0.05 as compared VLQ score at 3 months after treatment; @ *P <* 0.05 as compared VLQ score at 6 months after treatment

According to the VLQ questionnaire (Fig. 1), the baseline score of all patients was 2.490 ± 0.502, which was time-dependently improved with the treatment of temperature controlled dual-mode RF, accompanying by the higher score of 2.990 ± 0.696 at 1 month, 3.500 ± 0.909 at 3 months, 3.980 ± 1.090 at 6 months, and 4.441 ± 1.086 at 12 months (all *P <* 0.05). At 1 month, 3 months, 6 months and 12 months, there were 24.51% (25/102), 48.04% (49/102), 66.67% (68/102) and 79.41% (81/102) subjects reporting no VL (VLQ score ≥ 4), respectively, indicating the significant difference (*χ*^2^ = 169.9, *P <* 0.001). Most of the participants (92.16%, 94/102) reported an improvement in VLQ score of at least one point, with 71 of 102 subjects (69.61%) achieving improvement of two or more levels at 12 months.

### The improved sexual dysfunction in women after treated with temperature controlled dual-mode RF

**Fig. 2:**
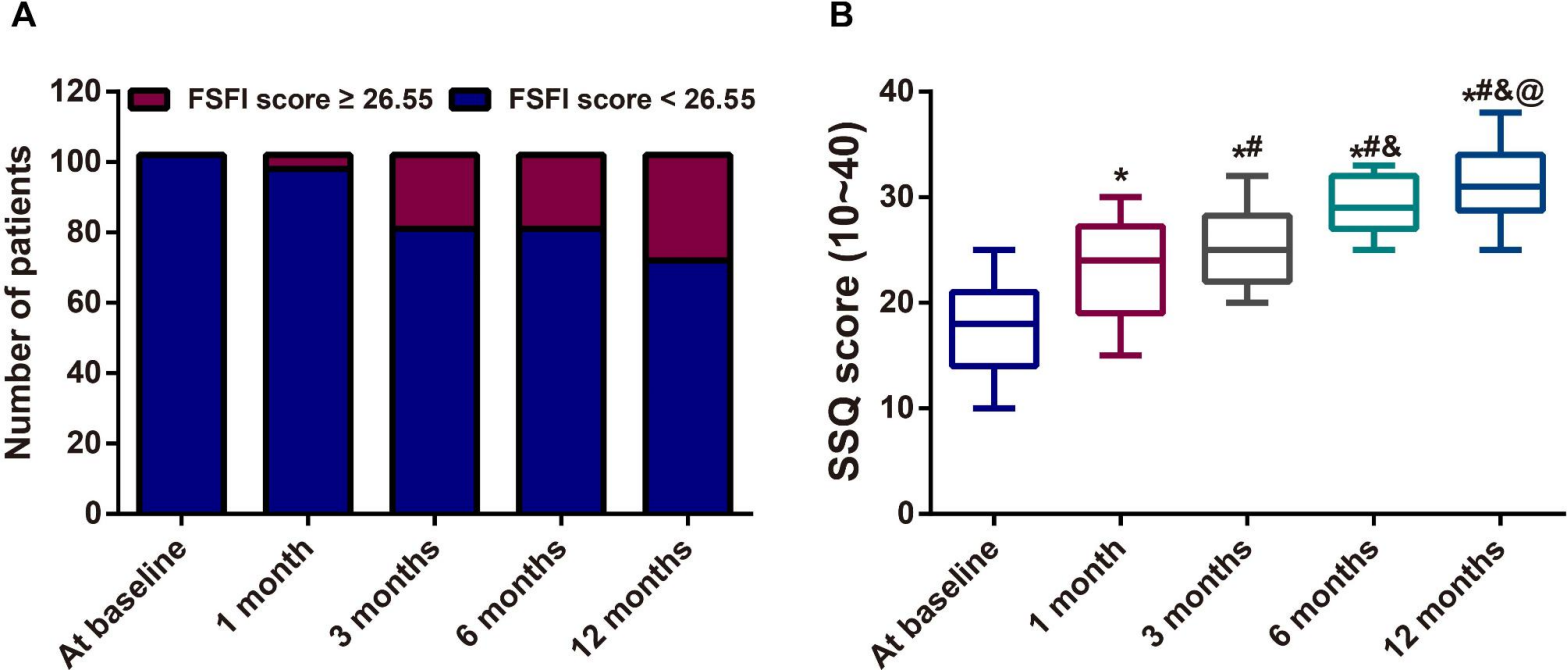
The improved sexual dysfunction in 102 women after temperature controlled dual-mode radiofrequency (RF) Note: A: Comparison of the proportion of subjects with the total Female Sexual Function Index (FSFI) score ≥ 26.55 at baseline, 1 month, 3 months, 6 months and 12 months; B: The increased sexual satisfaction was found after temperature controlled dual-mode RF treatment based on the Sexual Satisfaction Questionnaire (SSQ) score; * *P <* 0.05 as compared SSQ score at baseline; # *P <* 0.05 as compared SSQ score at 1 month after treatment; & *P <* 0.05 as compared SSQ score at 3 months after treatment; @ *P <* 0.05 as compared SSQ score at 6 months after treatment

Compared to the baseline of total FSFI score (< 26.55), statistically significant improvement was found at 1 month, 3 months, 6 months and 12 months after treated with temperature controlled dual-mode RF, as well as the six domains (sexual desire, sexual arousal, lubrication, orgasm, satisfaction, pain) in women (all *P <* 0.05, Table 3). All the scores of these six domains were increased at 6 months and 12 months when compared with those at 1 month (all *P <* 0.05). Overall, 29.41% of subjects (n = 30/102) experienced improvement from baseline in their combined measure of sexual functioning at 12 months with the total FSFI score ≥ 26.55 (Fig. 2A). Moreover, based on the SSQ score (10 ~ 40), the increased sexual satisfaction was found after the treatment with the temperature controlled dual-mode RF (*P <* 0.001, Fig. 2B).

**Table 3:** The improved sexual dysfunction in 102 women after temperature controlled dual-mode radiofrequency (RF) using Vaginal Laxity Questionnaire (VLQ) score

Six domains	At baseline	1 month	3 months	6 months	12 months
Sexual desire	3.069 ± 1.092	3.664 ± 0.908^*^	3.923 ± 0.822^*^	4.159 ± 0.718^*#^	4.289 ± 0.565^*#&^
Sexual arousal	2.702 ± 1.057	3.312 ± 1.003^*^	3.628 ± 0.830^*^	3.968 ± 0.732^*#&^	4.208 ± 0.623^*#&^
Lubrication	2.842 ± 1.137	3.479 ± 0.996^*^	3.822 ± 0.832^*#^	4.086 ± 0.689^*#^	4.273 ± 0.543^*#&^
Orgasm	2.669 ± 1.444	3.224 ± 1.307^*^	3.540 ± 1.193^*^	3.791 ± 1.073^*#^	4.009 ± 0.917^*#&^
Satisfaction	2.862 ± 1.177	3.407 ± 1.066^*^	3.754 ± 0.954^*^	3.984 ± 0.792^*#^	4.203 ± 0.667^*#&^
Pain	3.061 ± 1.206	3.546 ± 1.000^*^	3.862 ± 0.880^*^	4.121 ± 0.690^*#^	4.327 ± 0.546^*#&^
Total score	16.183 ± 4.414	20.631 ± 3.365^*^	23.675 ± 3.541^*#^	24.109 ± 2.348^*#^	25.309 ± 1.841^*#&@^

Note: * *P <* 0.05 as compared VLQ score at baseline; # *P <* 0.05 as compared VLQ score at 1 month after treatment; & *P <* 0.05 as compared VLQ score at 3 months after treatment; @ *P <* 0.05 as compared VLQ score at 6 months after treatment

### POP-Q examination findings in women with vaginal laxity after temperature controlled dual-mode RF

**Fig. 3:**
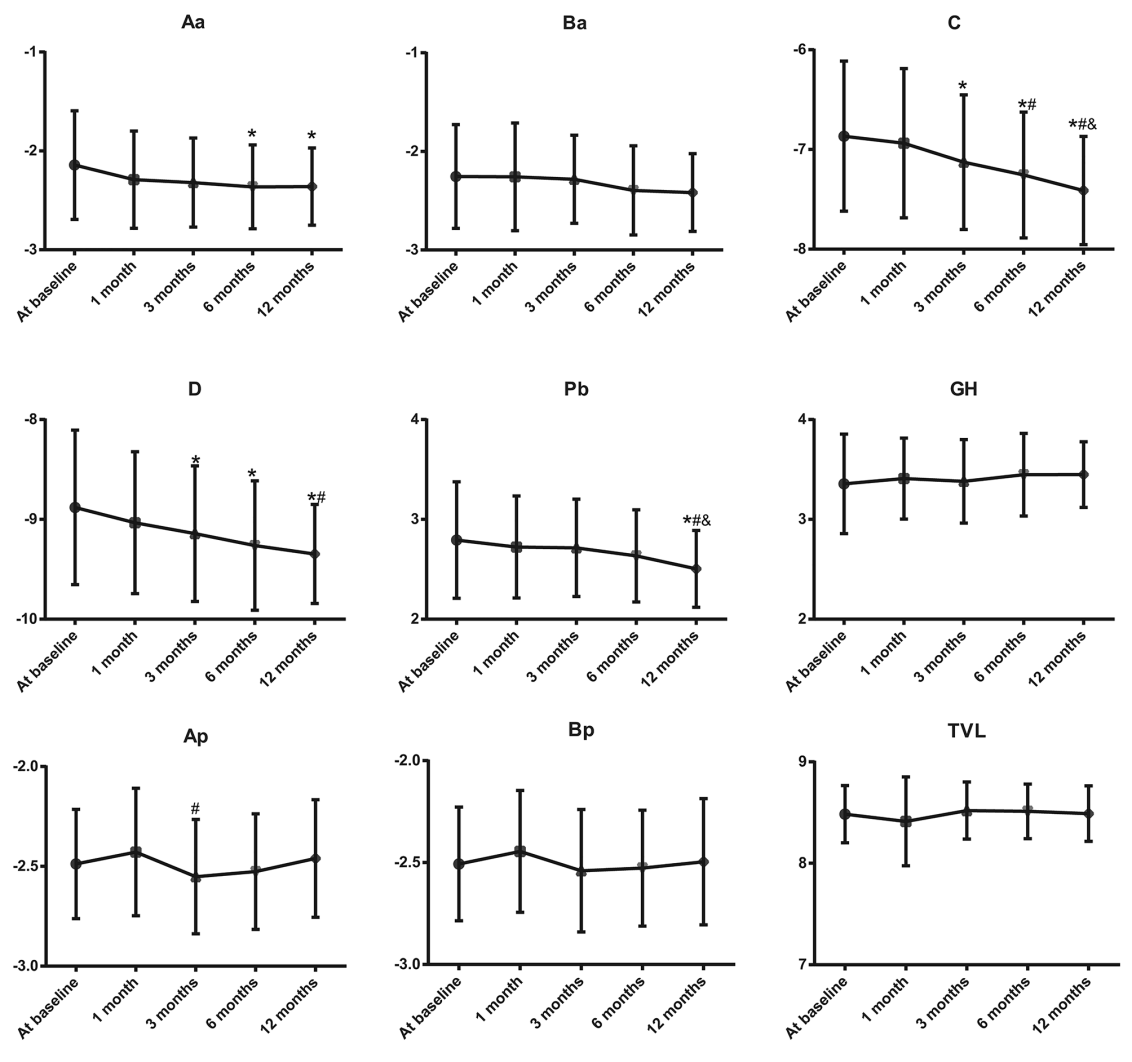
The examination findings of Pelvic Organ Prolapse Quantification System (POP-Q) in 102 women with vaginal laxity after temperature controlled dual-mode radiofrequency (RF), including point Aa, point Ba, point C, point D, perineal body (Pb), genital hiatus (GH), point Ap, point Bp and total vaginal length (TVL). Note: * *P <* 0.05 as compared the data at baseline; # *P <* 0.05 as compared the data at 1 month after treatment; & *P <* 0.05 as compared the data at 3 months after treatment; @ *P <* 0.05 as compared the data at 6 months after treatment

On vaginal examination, the POP-Q stage showed significant difference in women with vaginal laxity after temperature controlled dual-mode RF (*χ*^2^ = 36.79, *P <* 0.001), with the women having Stage I of 45.10 (46/102) at baseline, 36.27% (37/102) at 1 month, 28.43% (29/102) at 3 months, 19.61% (20/102) at 6 months and 10.78% (11/102) at 12 months. As illustrated in Fig. 3, the patients after temperature controlled dual-mode RF sessions demonstrated a lower Aa, point C, point D, and Pb at 12 months when compared the baseline data (all *P <* 0.05).

### The improved strength of voluntary contractions of the pelvic floor muscles in patients after temperature controlled dual-mode RF

In comparison with baseline data, the mean pressure and mean duration of pelvic contractions significantly increased in women at 1 month, 3 months, 6 months and 12 months after treatment (all *P <* 0.05, Table 4). Moreover, the mean pressure was significantly increased gradually at the 1-, 3-, 6- and 12- month follow-up (all *P <* 0.05). In addition, at 12 months follow-up, the longer duration of pelvic contractions in patients was found as compared with that at 3- and 6- months (all *P <* 0.05).

**Table 4:** The improved strength of voluntary contractions of the pelvic floor muscles in 102 patients after temperature controlled dual-mode radiofrequency (RF)

Time	Mean pressure (mmHg)	Duration (seconds)
At baseline	18.46 ± 2.83	5.19 ± 0.99
1 month	19.89 ± 3.05^*^	5.68 ± 1.06^*^
3 months	21.39 ± 3.11^*#^	6.19 ± 1.16^*#^
6 months	22.95 ± 3.36^*#&^	6.2 ± 1.18^*#^
12 months	23.94 ± 3.46^*#&@^	6.58 ± 1.44^*#&^

Note: * *P <* 0.05 as compared the data at baseline; # *P <* 0.05 as compared the data at 1 month after treatment; & *P <* 0.05 as compared the data at 3 months after treatment; @ *P <* 0.05 as compared the data at 6 months after treatment

## Discussion

RF treatments have been gaining popularity in the treatment of vaginal laxity due to the downtime and risk involved when compared to surgical procedures [31], offering an alternative for women who may not wish to undergo invasive surgical procedures [32]. In this study, after five times of RF therapy (monopolar mode for 15 min, bipolar mode for another 10-minute), 69.61% patients showed improvement in vaginal laxity according to VLQ score at 12 month and 29.41% patients obtained FSFI score no less than 26.55 at 12 months, accompanying by the elevated SSQ score, indicating temperature controlled dual-mode (monopolar and bipolar) RF contributed to the alleviated vaginal laxity and increased sexual satisfaction. Similarly, women completed the treatment of low-energy dynamic quadripolar RF showed improvements over the 12-month follow-up period in self-perception of introital looseness, related symptoms like dysuria/urinary incontinence, unrewarding sexual relationship, and atrophy-related symptoms (e.g., painful and unsatisfactory sexual activity) via assessing VLQ and SSQ scores [33]. A study using an RF device on the mucosal surface of the vaginal introitus of 30 women with self-reported vaginal laxity after giving birth showed significant and sustained improvements in their laxity, sexual function, and sexual distress for as long as 12 months of follow-up, with the higher FSFI total score at 12 months (26.0 ± 5.2) than the baseline data (22.4 ± 6.7) [32]. A monopolar RF device increased average FSFI of women undergoing once-a-week treatments after 12 months, indicating continuous RF treatment improved integrity at the vaginal introitus and sexual satisfaction [34]. However, Millheiser LS et al. demonstrated that the sexual arousal, orgasm experience, and satisfaction were improved after RF treatment for 6 months, but the increased lubrication was temporary, which returned to baseline at the 3-month follow-up [13]. The reasons might be a large disparity existing between treatment protocols and procedures, making the results difficult for determination of consistent effects.

Using POP-Q stage, we found significant improvement in pelvic floor function after treatment of dual-mode RF at 12 months. Besides, we also confirmed that dual-mode RF improved strength of voluntary contractions of the pelvic floor muscles, which was the most obvious at 12-month follow-up. The changes of vaginal fibroblast function and connective tissue components of vaginal wall may be related to POP [35], which could be improved by volumetric heating of vaginal tissue in the connective tissue organization [36]. RF therapy with a set temperature of 40 ~ 45 °C was reported to be correlated with vaginal elasticity recovery and increased vaginal mucosal moisture, and these actions were attributed to various factors, such as growth factor/collagen production in fibroblast via activating heat-shock proteins and inhibiting inflammatory cascade [21, 37]. Moreover, a histological analysis by Maia RR et al. showed fractional RF improved the number of fibroblasts, blood vessels, and fatty degeneration with higher type III collagen and vimentin expression [38]. Therefore, RF improved nerve sensitivity, vaginal vascularization, collagen fiber reorganization, thus contributing to a decrease in the sensation of vaginal laxity and an increased in sexual function, including arousal and orgasmic dysfunction [39, 40]. The mentioned above suggested the underlying mechanism of RA treatment to increase pelvic floor muscle strength.

As far as we know, for the first time, our study evaluated the efficacy and safety of temperature controlled dual-mode (monopolar and bipolar) RF regarding improvement of vaginal laxity and sexual function, as well as the strengthen of pelvic floor muscles. However, this study also has some limitations worth noting. Firstly, as promising alternative or adjunct treatments, non-surgical energy-based therapies and other noninvasive modalities have been proposed in women with sexual dysfunction, mainly including fractional microablative CO_2_ laser, erbium: YAG laser and temperature-controlled RF [41]. Fractional CO_2_ laser therapy was reported to be well tolerated by women with genitourinary syndrome of menopause and post-menopausal women with vaginal atrophy, which improved vaginal health, sexual functionality and quality of life [42, 43]. Moreover, a systematic review concluded that fractional CO_2_ laser treatment is an effective and safe therapeutic option for gynecological cancer survivors [44]. In addition, previous studies showed that sexual function was improved for 70–95% of patients with vaginal relaxation syndrome after erbium: YAG laser [45, 46]. However, we did not compare the efficacy and safety of fractional microablative CO_2_ laser, erbium: YAG laser and temperature-controlled RF in women with vaginal laxity. Further studies are required to determine whether temperature controlled dual-mode RF devices have any advantage over Er:YAG or CO_2_ lasers. Secondly, this is a retrospective study and not a randomized controlled trial, which should be further explored in the future with a relatively large number of patients. Finally, the generalizability of the study findings is restricted to women with self-reported vaginal laxity as defined by a baseline FSFI total score ≤ 26.55. Women who had higher baseline FSFI total score > 26.5 were not analyzed.

This study conducted to date have shown the improvement of vaginal laxity and sexual satisfaction, the reduction of POP risk, as well as the enhancement in pelvic floor muscles.

## Data Availability

All data generated or analysed during this study are included in this article.
